# High Quality 3D Photonics using Nano Imprint Lithography of Fast Sol-gel Materials

**DOI:** 10.1038/s41598-018-26261-3

**Published:** 2018-05-18

**Authors:** Ofer Bar-On, Philipp Brenner, Tobias Siegle, Raz Gvishi, Heinz Kalt, Uli Lemmer, Jacob Scheuer

**Affiliations:** 1 Department of Physical Electronics, Tel-Aviv University, Ramat-Aviv, 6997 Tel-Aviv, Israel; 2 Light Technology Institute, Karlsruhe Institute of Technology (KIT), 76131 Karlsruhe, Germany; 3 Institute of Microstructure Technology, Karlsruhe Institute of Technology (KIT), 76344 Karlsruhe, Germany; 4 Institute of Applied Physics, Karlsruhe Institute of Technology (KIT), 76131 Karlsruhe, Germany; 5 Photonic Materials Group, Applied Physics Division, Soreq NRC, 81800 Yavne, Israel

## Abstract

A method for the realization of low-loss integrated optical components is proposed and demonstrated. This approach is simple, fast, inexpensive, scalable for mass production, and compatible with both 2D and 3D geometries. The process is based on a novel dual-step soft nano imprint lithography process for producing devices with smooth surfaces, combined with fast sol-gel technology providing highly transparent materials. As a concrete example, this approach is demonstrated on a micro ring resonator made by direct laser writing (DLW) to achieve a quality factor improvement from one hundred thousand to more than 3 million. To the best of our knowledge this also sets a Q-factor record for UV-curable integrated micro-ring resonators. The process supports the integration of many types of materials such as light-emitting, electro-optic, piezo-electric, and can be readily applied to a wide variety of devices such as waveguides, lenses, diffractive elements and more.

## Introduction

Over the last few decades, integrated photonic devices have taken a dominant role in many fields such as communication, sensing, energy harvesting, and more. They offer superior features in terms of functionality, speed, accuracy, and show great promise for future applications. As a general rule, a technology for realizing high quality optical components must fulfil three basic requirements. First, a robust and highly transparent material is to be used. Second, the fabrication technique being used must be able to produce geometries with smooth surfaces, preferably versatile in 2D and 3D geometries. Finally, low cost and mass production compatibility are necessary in order to render such technology practical.

There is a wide variety of techniques, approaches and materials for the fabrication of integrated photonic devices. Standard UV lithography offers fast, accurate and cost-effective patterning but is usually limited to 2D geometries and suffers from roughness of the final device surfaces^1,2^. Another attractive and versatile fabrication techniques for 2D and 3D optical components is direct laser writing (DLW) - a 3D printing technique on the micron- and submicron scale^3,4^. Although this technique is very versatile in terms of geometry, it is still limited in terms of the process speed, the variety of materials it can pattern, and the attainable resolution due to the finite voxel size^5,6^. Although it is possible to overcome the first two limitations by combining DLW with soft nano imprint lithography (soft NIL)^7^, the roughness of the original master is retained and limits the performances of the final device. Soft NIL is a simple patterning method which enables the replication of structures fabricated using other methods^8–10^. Soft NIL is a fast and cost effective technique, maintaining the original geometry while offering a broader choice of materials for the replicated device. The great accuracy in which the original geometry is duplicated is also, in fact, a drawback of the method, as it also replicates the roughness of the original (master) device.

A common method for evaluating the impact of roughness is using quality factor (*Q*-factor) measurements of optical micro-ring resonators. Such measurements can quantify the propagation loss in the device (see characterization section below). Different groups have used direct thermal reflow of crosslinked SU-8 and other polymers in order to reduce the losses of waveguides and resonators^11,12^. However, the obtained *Q*-factors were lower than 10^5^. One of the most successful approaches for smoothening the surfaces of optical resonators was demonstrated by Vahala’s group utilizing high power CO_2_ lasers for inducing high temperature reflow of silica resonators^13^. Although this process was used for producing the highest known *Q*-factors for on chip micro-ring resonators, it is a very intense process which is difficult to precisely control. A slightly less intense roughness reduction step was demonstrated by Kalt’s group which realized high-Q reflowed polymer micro-resonators^14^. Nevertheless, both of the latter approaches are limited to the realization of specific geometries and cannot be readily applied as general and versatile tools for overcoming roughness in optical structures.

Thus, the problem of realizing smooth, highly transparent photonic devices with complex and diverse 2D and 3D geometries in a fast and cost effective manner still remains a major challenge. In this paper, we address this challenge by proposing and demonstrating a novel platform for realizing high quality, active and passive, photonic devices. The proposed process is based on transforming a prefabricated optical device (i.e. the master) into a more transparent and smoother version of itself. This is carried out by using a new fabrication approach designated as dual-step soft NIL (DSS-NIL) in combination with highly transparent sol-gel materials. DSS-NIL involves an intermediate low temperature reflow step which yields smoother surfaces than those of the master device. Fast sol-gel materials^15^ offer a robust and ultra-transparent materials to form the final high quality devices (more details on fast sol-gel in the methods section and supplementary material). This approach can be applied to various 2D or 3D devices and can pattern both passive and active materials. The method is fast, simple and inexpensive, thus satisfying all the requirements indicated above.

To demonstrate the extent of the improvement that can be achieved for a specific optical device, we employ this process to enhance the quality factor of an optical micro-resonator from one hundred thousand to more than 3 million. The substantial improvement in the Q-factor is accompanied by negligible change in the free spectral range (FSR), indicating that the general shape of the resonators has been maintained and that only the surface roughness was reduced. These results set a record for the Q-factor of UV-curable micro-resonators, raising it by an order of magnitude compared to previously published results^8^. (See supplementary material for more information).

## Results

### Dual Step Soft Nano Imprint Lithography

**Figure 1:**
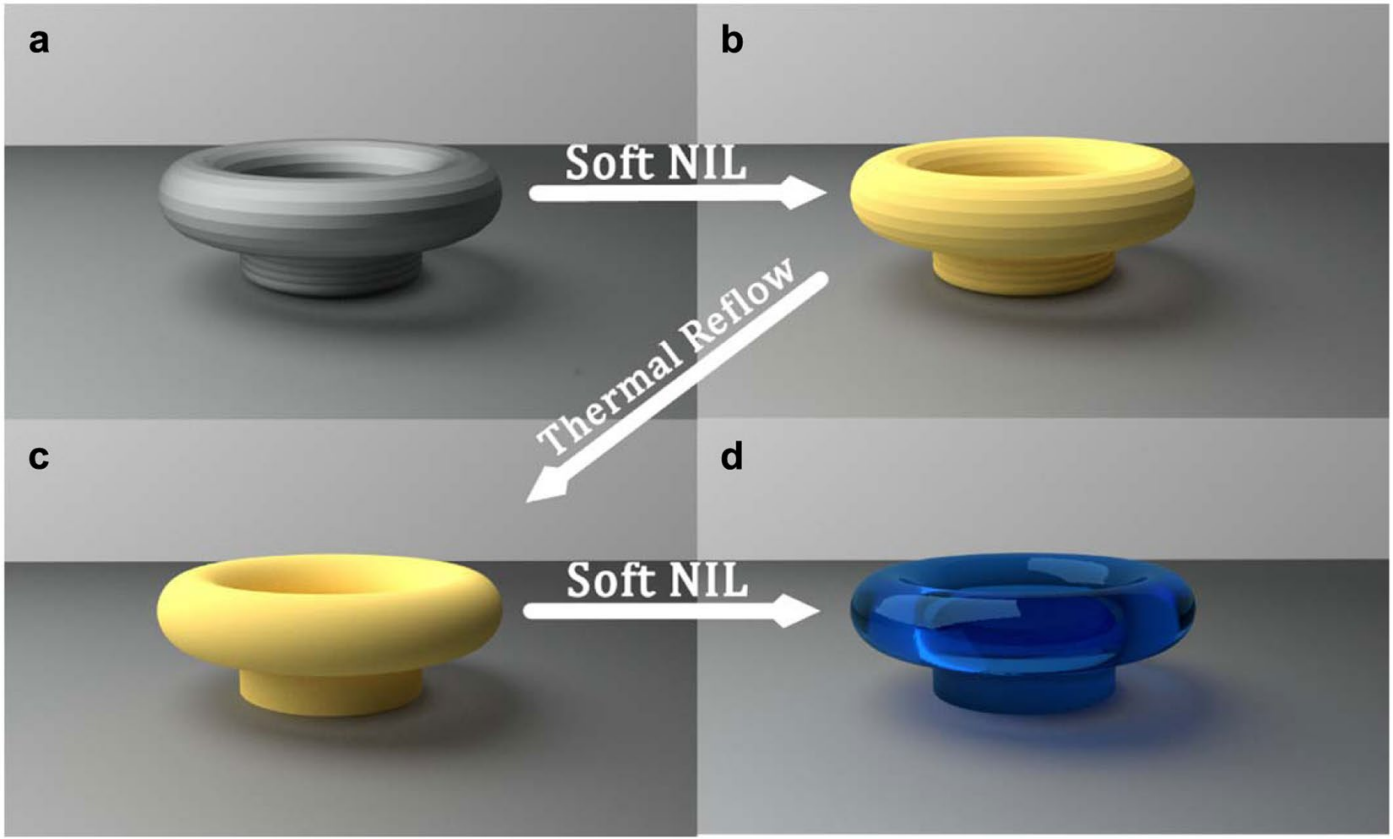
Dual-step soft nano imprint lithography process. (**a**) Master device fabricated using DLW made of crosslinked SU-8 (Grey). (**b**) First soft NIL step - Replication of the master device into non-crosslinked SU-8 (Yellow). (**c**) Reflowed replica from non-crosslinked SU-8 (Yellow). (**d**) Second soft NIL step - Replication of the reflowed resonator into sol-gel (Blue).

Standard soft NIL is a powerful process for the realization of various types of optical components. In this relatively simple process, an elastomeric material such as Polydimethylsiloxane (PDMS) is casted on a prefabricated template exhibiting the desired geometry of the final device. The PDMS is cured and peeled off from the master, forming a mold with the negative geometry of the master. This mold is now used to produce a replica of the master device by imprinting\molding. The fabrication process employed and reported on in this paper is based on a dual-step soft NIL process. The role of the first step is to eliminate the roughness of the device surfaces while the second step transforms the device into fast sol-gel. Combined together, the two steps yield devices with low surface roughness consisting of highly transparent material. The process flow is illustrated in Fig. 1.

A master device is fabricated by DLW (Fig. 1a), and replicated (Fig. 1b) using standard soft NIL into SU-8 without applying UV-illumination, thus, generating a non-crosslinked replica of the master. This is done by spin-coating a thin SU-8 film on a substrate followed by post baking. The substrate is heated above the SU-8 glass transition temperature while a soft PDMS mold (generated from the master device) is depressed upon it. Subsequently, the substrate is cooled to room temperature and the mold is released. This step produces a replica of the master consisting of non-crosslinked SU-8 which can be reflowed at relatively low temperatures (as opposed to the master fabricated by DLW which consists of crosslinked SU-8). Then, a low temperature thermal reflow step is carried out (Fig. 1c) to obtain a new version of the template with significantly reduced roughness. Finally, the reflowed structure is used as the new master for the realization of the UV-curable sol-gel device using a second standard soft NIL step (Fig. 1d). Note that using this method it is possible to reduce the surface roughness of a wide variety of optical components and transform them into improved materials. It should be noted that the reflow step can be carried out using long reflow times at low temperatures or using short reflow times at higher temperatures. Following a systematic optimization of the process we chose to work at lower temperatures (Between 35–40 °C) that are compatible with relatively long reflow times (~30 minutes) in order to render the process more repeatable. The proposed method is compatible with smaller/larger scale devices although slight modification of the reflow periods may be required.

**Figure 2:**
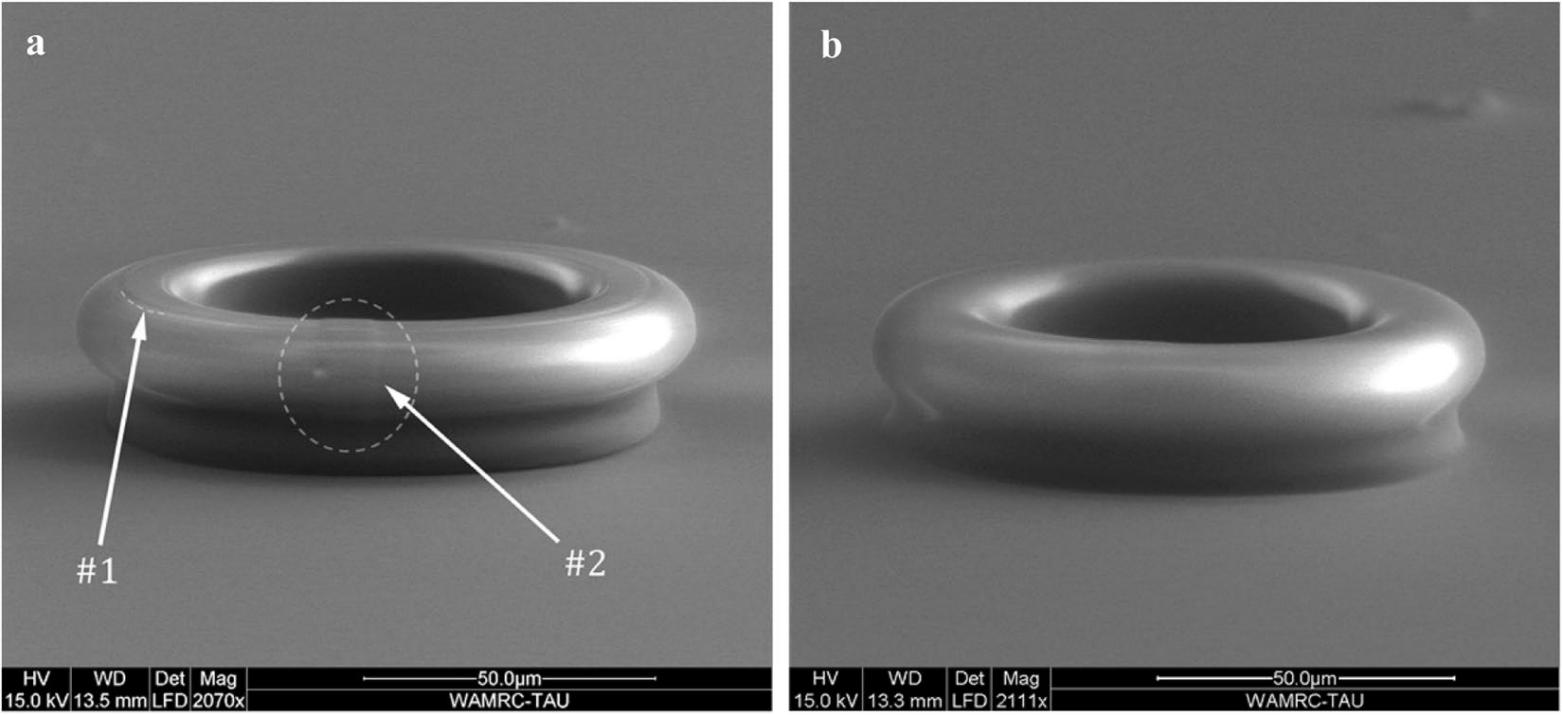
SEM images of sol-gel micro-resonators generated using (**a**) Standard soft NIL. (**b**) DSS-NIL. The numbers on figure ‘a’ point to defects from the original fabrication process. (1) Roughness resulted from the finite voxel size. (2) Bump resulted from the start stop point of the DLW process. As can be seen, both of these defects almost disappear entirely after the reflow process.

Figure 2 presents SEM images comparing sol-gel micro-resonators fabricated using standard soft NIL to devices fabricated by DSS-NIL. Figure 2a depicts the reference device, a 3D sol-gel micro-resonator, realized using standard soft NIL from a master device fabricated by DLW. Figure 2b, depicts a device realized using the DSS-NIL process. As can be seen in the images, the conventionally replicated device (without the reflow step) includes small artifacts that arise from the original fabrication method (DLW), such as rough edges from the finite voxel size (#1) and a small bump at the start-stop point of DLW writing process (#2).

On the other hand, the device that was fabricated using the improved DSS-NIL process is substantially smoother and, therefore, expected to exhibit lower propagation losses. Supplementary Figure 1S(a–d) shows SEM images of resonators fabricated using DSS-NIL at different reflow temperatures. When the reflow temperature reaches 40 degrees the general shape of the device begins to deform (The pedestal is diminishing). This can be controlled and greatly avoided by operating in the low temperature region or by pre-distorting the original device to compensate for the change. A more quantitative analysis of the losses reduction and the (potential) shape deformation of the structure due to the reflow process require detailed optical characterization of the devices. This analysis is detailed in the next section.

## Characterization

To demonstrate the virtues of the proposed process, we consider a concrete example and apply it to micro-ring resonators. In addition to their versatile functionality in numerous fields^16–19^, optical micro-resonators constitute an excellent tool for the characterization and evaluation of the fabrication process. More specifically, the quality factor, which can be evaluated directly from the spectral properties of the resonator^13,14^, is a direct measure of the propagation losses. The dominant loss mechanisms of properly designed circular resonators originate from surface roughness and material absorption. Thus, monitoring the quality factors of our ring resonators provides direct information on the ability of the fabrication process to reduce surface roughness and obtain transparent devices. In parallel, the free spectral range (FSR) is a direct measure of the resonator roundtrip or dimensions, thus implying on the general deformation of the structure during the reflow step.

**Figure 3:**
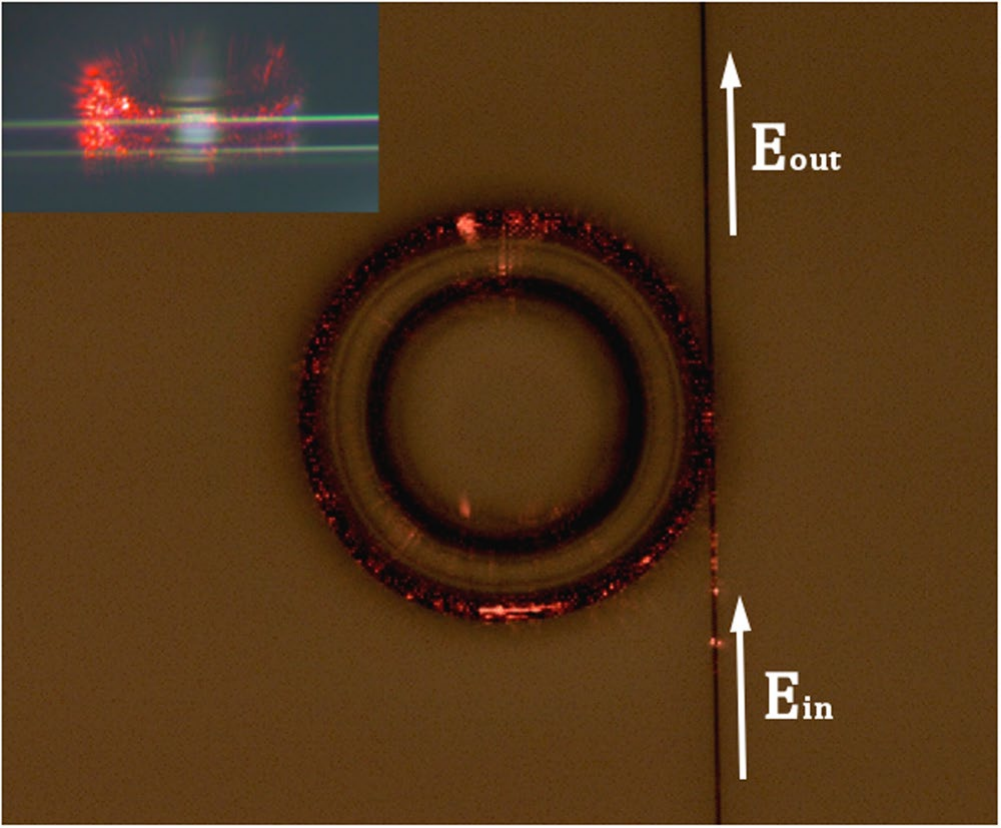
Light coupled into a non-reflowed (lossy) sol-gel micro-ring resonator in an ‘All Pass filter’ configuration from top view. Inset - side view.

**Figure 4:**
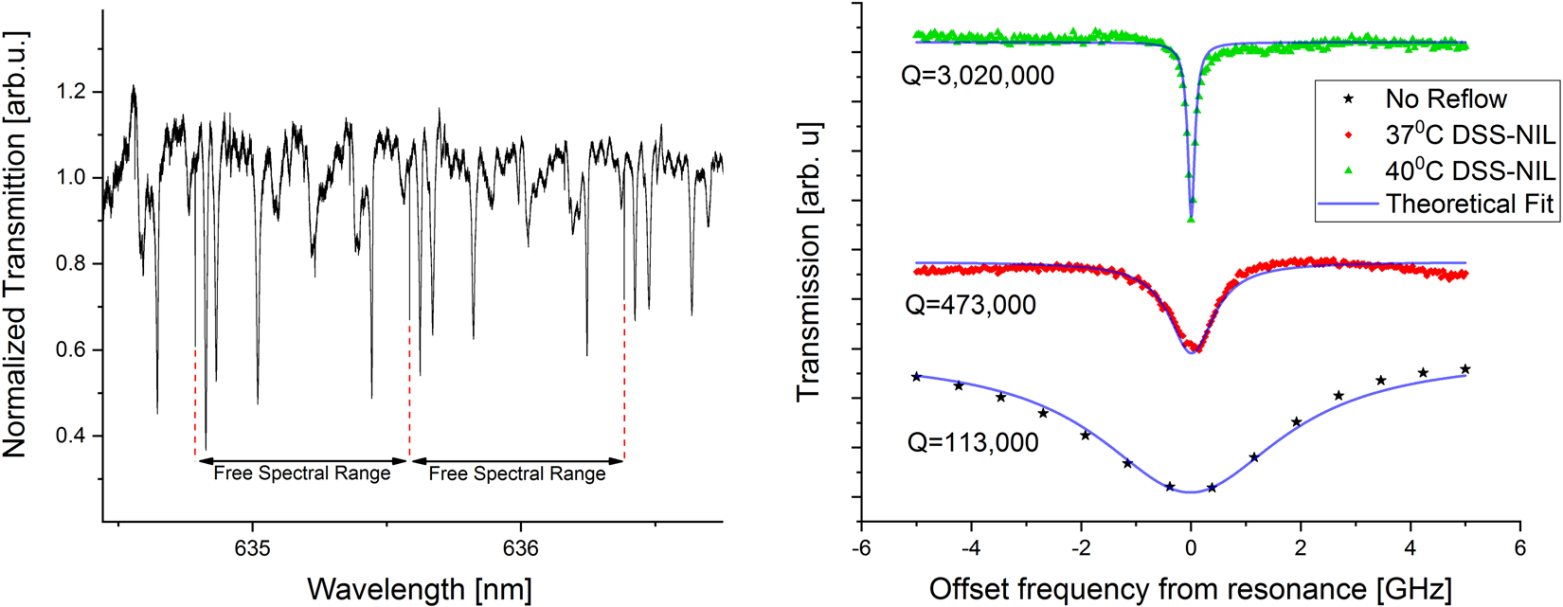
(**a**) Transmission spectra of a reflowed resonator. (**b**) Q factor comparison between resonators generated using standard NIL compared to DSS-NIL.

The characterization of the spectral properties is carried out by measuring the spectral transmission through a tapered fiber which is coupled to the resonator (this constellation is known as the all-pass filter configuration, see Fig. 3)^14^. This transmission of such configuration can be evaluated using^20,21^:

1 $$\frac{{E}_{out}}{{E}_{in}}=\frac{\sqrt{(1-\kappa )}-\sqrt{(1-\alpha )\,}{e}^{-i\frac{({\Delta }\omega )}{{\Delta }{\nu }_{FSR}}}}{1-\sqrt{(1-\kappa )(1-\alpha )}{e}^{-i\frac{({\Delta }\omega )}{{\Delta }{\nu }_{FSR}}}}$$

where *E*_*in*_ and *E*_*out*_ are respectively the amplitude of the input and output fields, Δ*ω* is the angular frequency shift from resonance, $\Delta {\nu }_{FSR}$ is the free spectral range, *κ* is the power coupling coefficient and *α* is the loss per revolution in the cavity. $\Delta {\nu }_{FSR}$ Can be extracted directly from the spectral transmission (Fig. 4a) while the coupling and loss coefficients can be obtained by fitting Eq. (1) to the measured spectral response (though without the ability to distinguish between the loss and coupling coefficients). Once these parameters are obtained, the *Q*-factor can be evaluated according to:

2 $$Q=\frac{{\omega }_{0}\sqrt{(1-\alpha )(1-\kappa )}}{2[1-\sqrt{(1-\alpha )(1-\kappa )}]{\Delta }{\nu }_{FSR}}$$

We note that Eq. (2) describes the *loaded Q*, which is a function of both the coupling coefficient and the loss in the cavity. The parameter which characterizes the loss properties of the cavity (and the quality of the process) is the *intrinsic quality factor, Q*_0_, corresponding to Eq. (2) where *κ* = 0. By varying the distance between the fiber and resonator it is possible to ensure that the cavity is critically (*Q*_0_ = 2*Q*) or strongly under coupled (*Q*_0_ ≈ *Q*). For properly designed micro-ring resonator, the bending/substrate leakage losses can be neglected and the quality factor of the device is determined primarily by the surface roughness and material absorption^22^:

3 $$\frac{1}{{Q}_{0}}\approx \frac{1}{{Q}_{abs}}+\frac{1}{{Q}_{scat}}$$

where *Q*_abs_ is the contribution of absorption to the loss and *Q*_scat_ is the scattering part which is due to surface roughness. This expression shows the relation between the *Q* factor and the roughness and absorption of the device. Figure 4b depicts a zoom in plot of the resonances obtained from the devices depicted in panels A (No reflow), C (reflow at 37 °C) and D (reflow at 40 °C) of Supplementary Figure 1S. As can be seen, the reflow step yields a significant narrowing of the resonance width indicating substantial improvement of the Q-factor and the propagation losses.

As indicated above, while the reflow step clearly improves the roughness of the device, an evaluation of the general deformation of the device geometry is also required. Such evaluation can be obtained by comparing the FSRs of devices that underwent different reflow steps. The FSR is defined as the distance between a two adjacent longitudinal modes and is related to the resonator geometry by the following expression:

4 $${\Delta }{\nu }_{FSR}=\frac{{c}_{0}}{2\pi R{n}_{g}}$$

Where *c*_0_ is the speed of light in vacuum, *n*_*g*_ is the group refractive index and *R* is the radius of the resonator, which indicates on any changes in the geometry of the device.

Table 1 summarizes the *Q*-factors and FSRs extracted from the characterization of resonators fabricated using different reflow process temperatures (due to phase mismatch, light cannot couple efficiently from the SiO_2_ fiber to the SU-8 master hence only sol-gel micro-resonators are detailed in the table). As can be seen, while the *Q* factor is monotonically improving with the reflow temperature (beyond an order of magnitude), the FSR (and hence the radius of the device) fluctuates with maximal variation below five percent.

**Table 1:** Parameters extracted from various resonators generated using different process parameters.

Extracted parameters	Standard soft NIL (No reflow)	36 (35.8) ^c^ Degrees [Celsius]	37 (36.9) Degrees [Celsius]	40 (40.3) Degrees [Celsius]
Loaded Q factor^a^	~110,000	~280,000	~470,000	~3,000,000
FSR^b^ [THz]	0.58	0.56	0.59	0.59

^a^Highest measured; ^b^Corresponding FSR.

^c^In parenthesis, the exact measured temperature.

These results clearly indicate that the presented approach can dramatically improve the surface roughness with only a minor impact on the device geometry. To the best of our knowledge, the resonator *Q* factors obtained by our process exceed the current state-of-the-art for UV curable resonators by an order of magnitude^8^. It should be noted that the maximal *Q* factor presented here is still limited by the surface roughness. The absorption limited *Q*-factor is determined by the propagation losses of FSG which, at the wavelengths investigated here, are approximately: 10^−3^ dB cm^−1^ ^15^. This value corresponds to a potential intrinsic quality factor of *Q*_0_ ~ 6 × 10^8^ indicating that there is still room for further roughness reduction.

## Discussion

In this paper we have demonstrated a new route towards the realization of optical components in 3D geometries, exhibiting low surface roughness. Combined with fast sol-gel technology, our approach provides the means for the realization of robust and arbitrarily-shaped devices, exhibiting low roughness surface and absorption. The utilization of NIL renders the process scalable and compatible with commercial applications. The approach can be employed to improve significantly the optical properties of existing devices or ones designed specifically for the process. Since the proposed approach is based on soft-NIL, its limitations in terms of material choice and geometry flexibility are similar to those of other NIL based techniques. Thus, based on previews studies utilizing soft-NIL, it is straightforward to apply it on organic dyes^7^ and electro-optic materials^23^ as well as piezo-electric materials^24^. In a similar manner, this approach is highly versatile in terms of geometry, limited mainly by the ability to mechanically release the soft mold^25,26^. Furthermore, it is compatible with direct laser writing, a true 3D digital fabrication approach for nano-photonics. As a concrete example, we employed the approach to demonstrate a UV curable micro-ring resonator with the highest Q-factor reported. It should be emphasized that in contrast to other thermal reflow process utilized for realizing high Q cavities, our approach is generic and suitable for obtaining a wide variety of optical devices requiring transparency and low roughness, e.g. diffractive elements, lenses, waveguides, and more. The combination of high transparency, low roughness and flexible doping render our approach very versatile and highly attractive for the realization of devices in many fields of optics.

## Methods

### Sol-gel preparation

The sol-gel materials were prepared using an automated PC-controlled apparatus which is located in a clean room environment. This apparatus allows mixing of quantitative amounts of precursors materials in a reactor cell, applying thermal heating in a sealed reactor cell and gradual pressure release. Figure 2S presents a temperature plot during the FSG process, for 10 consist runs, indicating the following steps; adding the precursors (constant temperature), increase in temperature due to exothermic reaction; increase in temperature above boiling temperature (70 °C) due to external heating and generation of pressure; decrease in temperature due to pressure release. Overall the process takes less than 20 min and the reproducibility of the process can be seen in Fig. 2S. In the current work, two recipes of FSG materials were produced: recipe 1 with molar ratio 1:6.5 of (TMOS:MTMS), designated as type “T” in ref.^27^, and recipe 2 with molar ratio of 1:5.6:0.4. (TMOS:MTMS:MAPTMS), designated as type “N” in ref.^27^. “T” type FSG can be cured only thermally while “N” type FSG can be cured either by thermally or by UV illumination. The UV-curing ability is due to the addition of small amount of the 3-methacryloxypropyltrimethoxysilane (MAPTMS) precursor which has three inorganic polymerizable tails (silicate) and one organic polymerizable tail (acrylic), where the acrylic tail can be UV-cured in the presence of a small amount of a photo-initiator. In this case we used a common photo-initiator, Irgacure 184 (4 wt%), which is colorless in the visible region, and was added to the final solution.

After mixing the precursors, the solution undergoes spontaneously hydrolysis and condensation reactions. In order to complete these reactions fast an acid catalyst is added, HCl, in two steps: ~1 × 10^−3^ molar together with the precursors and additional fraction, ~7.5 × 10^−5^ molar, after about 10 minutes. In addition, the process performed at a temperature of just below 100 °C where the reaction generates methanol and water vapors which induce pressure in the level of several atmospheres. Then the pressure is gradually releasing from several atmospheres to vacuum. In this way a viscous sol-gel resin (with viscosity of about 5,000 cPs) is quickly produced and can be solidified in a few minutes. In order to be able to use it for applications with a long shelf-life the viscose gel is immediately diluted by a tetrahydrofuran (THF) solvent at 1:1 weight ratio to obtain a solution with viscosity of ~5 cPs. The diluted FSG in THF solution can be kept in in a refrigerator for several months before use.

For NIL applications we used FSG in the following ways; In order to enable UV-curing process, “N type FSG” solution was used where a small quantity of the photo-initiator was added to the diluted FSG in THF solution.

### Master Device Fabrication

The master micro-resonators templates were fabricated using direct laser writing as described in previews studies^7,28^.

### DSS-NIL

The master device was coated with a few nanometers of C4F8 to serve as anti-adhesive coating. This step was followed by casting commercial PDMS (Sylgard 184) mixed with its curing agent in a ratio of 10:1 on the sample. The PDMS was cured at room temperature for at least 3 days and peeled from the master device to serve as a mold. Then, a layer of SU-8 3005 was spin coated on top of a Si substrate at a spin rate of 5000 rpm followed by 3 minutes of soft baking on a hot plate at 95 °C. The first soft NIL step was then carried by pressing the mold on the substrate at elevated temperature followed by cooling. Detailed imprint process parameters are presented in chapter 4 of the supporting information. Finally, the mold was released yielding a non-crosslinked replica of the master device (Since no UV-illumination was applied). This replica was placed in a convection bake oven for the reflow process while a thermometer was placed right next to the sample in order to monitor the exact temperature of the process. The samples were kept in the oven for 30 minutes under the reflow temperature. These reflowed devices were used as masters for the next soft NIL step. Similar to the original master preparation process discussed above, the new master was coated with anti-adhesive coating and a PDMS mold was generated from it. For the second soft NIL step, UV curable sol-gel was spin coated on a Si substrate at a spin rate of 5000 RPM and placed on a 60 degrees hot plate for one minute in order to evaporate the solvent. The sample was then placed inside the NIL machine and was patterned using the mold generated from the reflowed sample. The detailed imprint process parameters are presented in chapter 4 of the supporting information. The mold was then released, leaving a smooth and transparent sol-gel replica of the master device.

## Electronic supplementary material

Supplementary information
