# Supplementary material for: High Quality 3D Photonics using Nano Imprint Lithography of Fast Sol-gel Materials
Source: Sci Rep. 2018 May 18;8:7833. doi: 10.1038/s41598-018-26261-3 (PMC5959872; doi:10.1038/s41598-018-26261-3)
Supplement: Supplementary file 1 — Supplementary information [file 41598_2018_26261_MOESM1_ESM.docx]

Supporting Information

Title High Quality 3D Photonics using Nano Imprint Lithography of Fast Sol-gel Materials

Ofer Bar-On, Philipp Brenner, Tobias Siegle, Raz Gvishi, Heinz Kalt, Uli Lemmer, and Jacob Scheuer*

1. **Micro-ring resonators generated by imprinting/molding in the last years**

In the last two decades, various groups have demonstrated different methods for the realization of micro-ring resonators, due to its importance to the field of integrated photonics. Several molding/imprinting techniques were used for the task to offer simple, fast and inexpensive implementation. The table below presents a short summary of micro ring resonators generated by molding / imprinting.

| **Ref.** | **Description** | **NIL type** | **Year** | **Q-Factor** |
| --- | --- | --- | --- | --- |
| ^[1]^ | Very high Q-factors were demonstrated in this work using molding with thermal curing. However, the process demonstrated in that paper requires approximately 60 hours of curing which limits the speed of the process. In addition, the mold in this work was based on a very specific template fabrication approach. | Thermal | 2004 | $5*{10}^{6}$ |
| ^[2]^ | The current record for UV curable micro ring resonators. | UV | 2007 | $3*{10}^{5}$ |
| ^[3]^ | Demonstrated pressure sensors based on planar micro ring resonators generated using improved nano-imprint lithography technique. | Thermal | 2011 | ${10}^{5}$ |
| ^[4]^ | Demonstrated low residual layer planar micro ring resonators using direct UV imprinting. | UV | 2014 | $1.3*{10}^{4}$ |
| ^[5]^ | Thermal nano-imprint lithography of Chalcogenide glass. | Thermal | 2014 | $4*{10}^{5}$ |
| ^[6]^ | Demonstrated planar micro ring resonators for the near infra-red region using UV nano imprint lithography | UV | 2016 | $3.9*{10}^{4}$ |

1. **DSS-NIL under different process parameters**











d

c

b

a

Figure 1S. Sol-gel micro resonators generated using different reflow step temperatures. a) No reflow. b) DSS-NIL at 36^0^C. c) DSS-NIL at 37^0^C. d) DSS-NIL at 40^0^C.

1. Fast sol-gel

The sol-gel process is a well-known chemical route to produce glassy based materials at low temperatures.^[7,8]^ The two main advantages of the sol-gel process are (1) the ability to produce versatile final configurations such as monolith/bulk glasses, thin films/coatings, nanoparticles, fibers, and other arbitrary geometries; and (2) the ability to incorporate in the sol-gel matrix a variety of organic/inorganic functional additives. The process includes three main steps; the formation of a sol (hydrolysis), gelation of the sol (condensation) and removal of the solvent. The chemical reactions which describe the sol-gel process are:

$Hydrolysis: nSi(OR)4+4nH2O \boldsymbol{\leftrightarrow} nSi(OH)4 + 4nROH$ (1)

$Condensation: nSi(OH)4 \boldsymbol{\to} nSiO2 + 2nH2O$

The sol-gel process enables the growth of a three-dimensional glass-like network with controlled properties depending on the composition and process conditions. The obtained material is very robust and transparent, its refractive index can be tuned, and it is solution processable, thus rendering it inexpensive and simple to apply. Therefore, sol-gel technology can be considered as a highly attractive “tool kit” for the preparation of glassy and ceramic materials including organic-inorganic hybrid materials. Sol-gel materials have been used for various applications such as miniature lenses,^[9]^ protective and functional coatings,^[10]^ anti-reﬂection,^[11]^ anti-scratch,^[12]^ fog and contamination coatings.^[13]^ Sol-gels are also used as ceramic powders,^[14]^ in the fabrication of optical ﬁbers,^[15]^ micro-cavities,^[16]^ and Bragg reflectors.^[17]^ Recently, several studies demonstrated nanoimprint lithography (NIL) with hybrid sol-gel materials.^[18–21]^

In recent years, upgraded sol-gel technologies have emerged such as fast sol-gel (FSG), which overcome the drawbacks of the original method such as material shrinkage and tendency to crack. These improvements render FSG even more suitable for applications in various fields.^[22]^ In the past, we have presented a class of sol-gel materials prepared in the FSG process.^[22,23]^ Such materials are solidified using thermal or UV-curing and exhibiting thermal stability over 300ºC, adhesive strength exceeding 10MPa and excellent transparency from 400-1100 nm (losses below 0.01 dB/cm).^[24]^ The FSG process allows the preparation of crack-free sol–gel materials without shrinkage with low residual organic content (<20 wt%). The method facilitates the fabrication of a viscous sol–gel resin in a few minutes followed by either thermal-curing (at 65ºC) in several hours or UV-curing in several seconds,^[24]^ thus rendering it highly applicable for the soft NIL fabrication approach presented here. (More information on FSG, background and preparation procedure can be found in the supplementary and experimental sections)

Fast sol–gel is distinguished by the short time required to achieve a solid monolith. The fast sol–gel can be cast either as a bulk monolith, thin film (submicron to a few hundred microns) or as a bonding phase between glass components^[25,26]^. Recently we demonstrated the use of FSG material as matrix for optical devices prepared by soft NIL^[27]^. This class of materials is a promising candidate for preparation of optical elements such as waveguides and submicron structured replicas and can also be used as an optical bonding material. The FSG method uses a combination of organically modified alkoxides with traditional alkoxides as precursors, to produce a final product which is an organic-inorganic hybrid with properties that can vary from silicone rubbers to silica glass. Optical and physical properties, such as refractive index and thermal expansion, can be engineered by controlling the ratio between the precursors^[28]^. The concept of the fast sol–gel process is to complete the reaction of all the precursors and evacuation of the unnecessary products (alcohol and water) before gelation occurs. The process starts without adding any additional alcohol solvent and since the first stage is performed in a closed vial, the alcohol which is produced in the reaction acts as a common solvent. The procedure involves mixing of alkoxide and organically modified alkoxide precursors, such as Tetramethoxysilane (TMOS), Methyltrimethoxysilane (MTMS) and Dimethoxydimethylsilane (DMDMS), in a reaction vessel. An example of fast sol–gel starting combination is a mixture containing TMOS:MTMS:H_2_O in molar ratio 0.2:1:2, respectively. The system is kept closed, allowing the temperature and pressure to increase without boiling above the standard boiling temperature of the alcohol, until all the hydrolysis of the precursors is completed. Then, a rapid evacuation of the unnecessary products (alcohol and water) is performed by releasing the pressure and pumping the system below atmospheric pressure. The process is stopped before gelation occurs, where the gel contains about 4% residual liquid and the material is still in a solution state. The product at this stage is a viscous resin which can be poured into a mold or diluted for long shelf-life storage.





**Figure 2S.** Temperature plot during the FSG process, for 10 consist runs, indicating the following steps; adding the precursors (constant temperature), increase in temperature due to exothermic reaction; increase in temperature above boiling temperature (70ºC) due to external heating and generation of pressure; decrease in temperature due to pressure release.

1. NIL recipes

**SU-8-Imprint (First step)**

| Temperature [C] | Time to reach temperature [sec] | Time to apply temperature [sec] |
| --- | --- | --- |
| 100 | 10 | 125 |
| *Room temperature* | 315 | 0 |

| Pressure [Kg cm^-2^] | Time to reach pressure [sec] | Time to apply pressure [sec] |
| --- | --- | --- |
| 5 | 60 | 40 |
| 0.8 | 30 | 120 |

**Sol-gel imprint (Second step)**

| Temperature [C] | Time to reach temperature [sec] | Time to apply temperature [sec] |
| --- | --- | --- |
| 65 | 50 | 50 |
| 100 | 50 | 450 |
| *Room temperature* | 300 | 0 |

| Pressure [Kg cm^-2^] | Time to reach pressure [sec] | Time to apply pressure [sec] |
| --- | --- | --- |
| 1 | 100 | 800 |

| UV @365nm [mW/cm^2^] | Time to apply UV [sec] |
| --- | --- |
| 0 | 250 |
| 132 | 650 |

References:

[1] A. L. Martin, D. K. Armani, L. Yang, K. J. Vahala, *Opt. Lett.* **2004**, *29*, 533.

[2] A. M. Armani, A. Srinivasan, K. J. Vahala, *Nano Lett.* **2007**, *7*, 1823.

[3] T. Ling, S.-L. Chen, L. J. Guo, *Opt. Express* **2011**, *19*, 861.

[4] R. Kirchner, A. Finn, R. Landgraf, L. Nueske, L. Teng, M. Vogler, W.-J. Fischer, *J. Light. Technol.* **2014**, *32*, 1674.

[5] Y. Zou, D. Zhang, H. Lin, L. Li, L. Moreel, J. Zhou, Q. Du, O. Ogbuu, S. Danto, J. D. Musgraves, K. Richardson, K. D. Dobson, R. Birkmire, J. Hu, *Adv. Opt. Mater.* **2014**, *2*, 478.

[6] R. Morarescu, P. K. Pal, N. T. Beneitez, J. Missinne, G. V. Steenberge, P. Bienstman, G. Morthier, *IEEE Photonics J.* **2016**, *8*, 1.

[7] G. W. S. C.J. Brinker, *The Physics and Chemistry of Sol Gel Processing*, Academic Press Inc., San-Diego, **1990**.

[8] R. Gvishi, in *Sol-Gel Handb. — Synth. Charact. Appl.*, Wiley-VCH, Weinheim, Germany, **2015**.

[9] J. Rogers, A. Kärkkäinen, T. Tkaczyk, J. Rantala, M. Descour, *Opt. Express* **2004**, *12*, 1294.

[10] S. Sultan, K. Kareem, L. He, *Surf. Coatings Technol.* **2016**, *300*, 42.

[11] A. Jannat, W. Lee, M. S. Akhtar, Z. Y. Li, O. B. Yang, *Appl. Surf. Sci.* **2016**, *369*, 545.

[12] H. Lee, C. K. Yao, J. Der Liao, P. L. Shao, M. H. N. Thi, Y. H. Lin, Y. Der Juang, *Mater. Des.* **2015**, *88*, 651.

[13] W. Huang, Y. Chen, C. Yang, Y. Situ, H. Huang, *Ceram. Int.* **2015**, *41*, 7573.

[14] M. Chandrasekhar, P. Kumar, *Ceram. Int.* **2016**, *42*, 10587.

[15] A. Chahadih, H. El Hamzaoui, O. Cristini, L. Bigot, R. Bernard, C. Kinowski, M. Bouazaoui, B. Capoen, *Nanoscale Res. Lett.* **2012**, *7*, 487.

[16] J. Park, S. K. Ozdemir, F. Monifi, T. Chadha, S. H. Huang, P. Biswas, L. Yang, *Adv. Opt. Mater.* **2014**, *2*, 711.

[17] B. Brudieu, A. Le Bris, J. Teisseire, F. Guillemot, G. Dantelle, S. Misra, P. R. i. Cabarrocas, F. Sorin, T. Gacoin, *Adv. Opt. Mater.* **2014**, *2*, 1105.

[18] W. S. Kim, J. H. Lee, S. Y. Shin, B. S. Bae, Y. C. Kim, *IEEE Photonics Technol. Lett.* **2004**, *16*, 1888.

[19] G. Brusatin, G. Della Giustina, *J. Sol-Gel Sci. Technol.* **2011**, *60*, 299.

[20] L. Brigo, G. Grenci, A. Carpentiero, A. Pistore, M. Tormen, M. Guglielmi, G. Brusatin, *J. Sol-Gel Sci. Technol.* **2011**, *60*, 400.

[21] G. Della Giustina, G. Brusatin, M. Guglielmi, C. Palazzesi, E. Orsini, P. Prosposito, *Solid State Sci.* **2010**, *12*, 1890.

[22] R. Gvishi, *J. Sol-Gel Sci. Technol.* **2009**, *50*, 241.

[23] T. Hanuhov, E. Asulin, R. Gvishi, *J. Non. Cryst. Solids* **2017**, *471*, 301.

[24] R. Gvishi, A. Englander, G. Strum, *J. Eur. Opt. Soc.* **2012**, *7*, 1.

[25] R. Gvishi, G. Strum, N. Shitrit, R. Dror, *Opt. Mater. (Amst).* **2008**, *30*, 1755.

[26] R. Gvishi, M. Pokrass, G. Strum, *J. Eur. Opt. Soc.* **2009**, *4*, 1.

[27] P. Brenner, O. Bar-On, T. Siegle, T. Leonhard, R. Gvishi, C. Eschenbaum, H. Kalt, J. Scheuer, U. Lemmer, *Appl. Opt.* **2017**, *56*, 3703.

[28] M. Pokrass, Z. Burshtein, R. Gvishi, *Opt. Mater. (Amst).* **2010**, *32*, 975.
